# The impact of anthropogenic noise on individual identification via female song in Black-capped chickadees (*Poecile atricapillus*)

**DOI:** 10.1038/s41598-021-96504-3

**Published:** 2021-09-02

**Authors:** Carolina Montenegro, William D. Service, Erin N. Scully, Shannon K. Mischler, Prateek K. Sahu, Thomas J. Benowicz, Katelyn V. R. Fox, Christopher B. Sturdy

**Affiliations:** 1 Department of Psychology, University of Alberta, Edmonton, AB Canada; 2 University of Alberta, Neuroscience and Mental Health Institute, Edmonton, AB Canada

**Keywords:** Behavioural ecology, Animal behaviour

## Abstract

When anthropogenic noise occurs simultaneously with an acoustic signal or cue, it can be difficult for an animal to interpret the information encoded within vocalizations. However, limited research has focused on how anthropogenic noise affects the identification of acoustic communication signals. In songbirds, research has also shown that black-capped chickadees (*Poecile atricapillus*) will shift the pitch and change the frequency at which they sing in the presence of anthropogenic, and experimental noise. Black-capped chickadees produce several vocalizations; their *fee-bee* song is used for mate attraction and territorial defence, and contains information about dominance hierarchy and native geographic location. Previously, we demonstrated that black-capped chickadees can discriminate between individual female chickadees via their *fee-bee* songs. Here we used an operant discrimination go/no-go paradigm to discern whether the ability to discriminate between individual female chickadees by their song would be impacted by differing levels of anthropogenic noise. Following discrimination training, two levels of anthropogenic noise (low: 40 dB SPL; high: 75 dB SPL) were played with stimuli to determine how anthropogenic noise would impact discrimination. Results showed that even with low-level noise (40 dB SPL) performance decreased and high-level (75 dB SPL) noise was increasingly detrimental to discrimination. We learned that perception of *fee-bee* songs does change in the presence of anthropogenic noise such that birds take significantly longer to learn to discriminate between females, but birds were able to generalize responding after learning the discrimination. These results add to the growing literature underscoring the impact of human-made noise on avian wildlife, specifically the impact on perception of auditory signals.

## Introduction

Throughout the world, the anthropogenic pressures of human activity, including anthropogenic noise, are increasing and have significant effects on animal behavior^1,2^. Anthropogenic noise consists of a wide range of sounds including: road vehicles, airplanes, industrial machinery, and air movement machinery^3,4^. Anthropogenic noise levels have been shown to cause disturbances in human and non-human animals depending on the source and its proximity^5^. In non-human animals, the disturbances associated with exposure to long-term anthropogenic noise include physical and/or physiological damage, and masking of communication signals^5,6^.

The vocal adjustments made by songbirds as a consequence of anthropogenic noise are currently well studied. For example, songbirds can shift their temporal pattern of behaviour by singing earlier in the day to avoid noises associated with high traffic noise^7,8^, alter the quality of their vocalizations by shifting frequency or amplitude^9^, and change frequency and duration as a consequence of anthropogenic noise exposure^10^. Killer whales (*Orcinus orca*) will increase call duration in the presence of anthropogenic noise^11^, while bow-winged grasshoppers (*Chorthippus biguttulus*) will increase the frequency of courtship signals in response to increasing anthropogenic noise^12^.

The auditory masking of signals and its influences on perception of acoustic signals have been demonstrated in a variety of species. In the presence of traffic noise, great tit (*Parus major*) alarm calls are masked, and thus a decrease in anti-predator responses can be observed^13^. Similarly, another study found that when exposed to playbacks of tufted titmouse (*Baeolophus bicolor)* alarm calls in noisy areas, cardinals (*Cardinalis cardinalis*) were less likely to produce predator avoidance behaviors compared to behavior in quieter areas suggesting the ability to eavesdrop had decreased due to noise^14^. A comparable impact has been demonstrated in hermit crabs (*Coenobita clypeatus*), in this study, crabs were exposed to boat motor playbacks. In the presence of the motor noise, crabs were slower to hide in response to a simulated visual predator, but, when adding a flashing light, crabs took even longer to hide, suggesting that the noise distracted the crabs from hiding in their shells (distracted prey hypothesis)^15^. Anthropogenic noise in the form of traffic noise has also been shown to impact grey treefrogs (*Hyla chrysoscelis*) ^16^. In this particular study, female treefrogs were presented with varying levels of male advertisement calls, these calls are produced by males in order to attract females. Results showed a similar increase in response latency and decrease in orientation by females towards male calls when in the presence of a simulated breeding chorus or when in the presence of simulated road traffic noise, suggesting that anthropogenic noise masks a female's perception of male calls in a similar way to a breeding chorus.

Similarly, there is ample research on vocal adjustment in black-capped chickadees, which includes several studies on masking of vocal signals, but few on the perception of masked signals. The black-capped chickadee (*Poecile atricapillus*) *fee-bee* song is a two-note vocalization that is used primarily by males for territorial defense and mate attraction^17,18^. Prior research has shown that black-capped chickadees will shift the pitch^22^ and frequency^23^ at which they sing in the presence of anthropogenic noise. Furthermore, anthropogenic noise can mask acoustic signals and compromise discrimination of fine details in songs in both great tits (*Parus major*), and blackbirds (*Turdus merula*)^24,25^.

In order to avoid wasting resources (i.e., time and energy), distinguishing between individuals is a useful ability. We can observe the ability to identify individuals by their vocalization in several species including but not limited to conspecifics in song sparrows (*Melospiza melodia*)^26^ and with mates in zebra finches (*Taeniopygia guttata*)^27^. Most recently for black-capped chickadees, the *fee-bee* song has been shown to serve a function related to individual recognition in terms of mate recognition, in particular the *fee* glissando^28^. A past bioacoustic analysis revealed that the *fee* glissando (decrease in frequency compared to the first *fee*-note and following *bee-*note)^29^ is less prominent in male chickadees compared to female chickadees^28^. A follow-up operant conditioning go/no-go task found that the both sexes of chickadee can discriminate between female and male *fee-bee* songs, thus suggesting that the *fee* glissando is an acoustic feature used for identification^28,30^. Furthermore, past song studies have suggested that features such as total song duration and the interval ratio are useful in discrimination between males^31^. The above features may also indicate male quality, especially if we consider that chickadees only have one song with two functions: mate attraction and territorial defense. In addition, other features such as relative amplitude of the two notes demonstrate significant differences between dominant and subordinate male chickadees^32^. Accordingly, song may be used to tell individuals apart based on sex, quality, and rank.

It has long been known that in tropical species female song exists as a function of duetting with male mates^19,33^; however, an increasing number of studies support the notion that in many temperate songbird species, females also sing, thus creating a geographic bias for reporting and investigating which female songbirds sing^34,35^. Recent research surrounding female song in other songbirds has suggested that the function of female song is most likely similar to the functions of male song^19,21,35^,, that is, territorial defense and mate attraction. Specifically, in black-capped chickadees, the proposed function of female song is mate recognition^28^. Previous research suggests that female song does differ from male song^28^, and females and males can discriminate between female- and male-produced songs^30^. Recently, we have also shown that male and female black-capped chickadees can discriminate between individual females via their *fee-bee* songs^36^.

Based on our own findings and the findings of current anthropogenic noise literature, we questioned whether chickadees could discriminate among female songs in the presence of anthropogenic noise. We used an operant go/no-go paradigm to determine how anthropogenic noise impacts the ability of male and female black-capped chickadees to discriminate between individual female black-capped chickadee *fee-bee* songs. Male and female black-capped chickadees were trained and tested using unmanipulated *fee-bee* songs in addition to varying levels of anthropogenic noise. Our aim was to examine if the chickadees could identify individual female chickadees via their *fee*-*bee* song, as previously found, at differing levels of anthropogenic noise measured on how quickly the birds were able to learn to distinguish between individuals. We predict that noise will be detrimental to learning this discrimination at all levels and stages of the current study with low level noise being less detrimental compared to high level noise, thus performance (i.e., number of trials to learn the discrimination) will be fewest (best) during no noise.

## Methods

### Subjects

In total, twenty-two black-capped chickadees (nine males and 13 females) were tested between May and December 2019, and 16 black-capped chickadees (seven males and nine females) completed the experiment. One male and one female failed to learn Pretraining, and one female failed to learn Non-differential training (see descriptions below for training information); as a result, all three were removed from the experiment. In addition, one male and two females died of natural causes during the course of the study (see Ethics Declaration). For all birds, sex was determined by deoxyribonucleic acid analysis of blood samples^37^. All birds were captured in Edmonton (North Saskatchewan River Valley, 53.53°N, 113.53°W; Mill Creek Ravine, 53.52°N, 113.47°W), Alberta, Canada in January 2018 and January 2019 and were at least one year of age at capture, verified by examining outer tail rectrices^38^.

Prior to the current experiment, all chickadees were individually housed in Jupiter Parakeet cages (30 × 40 × 40 cm; Rolf C. Hagen, Inc., Montreal, QB, Canada) in a single colony room. Therefore, birds did not have physical contact with each other, but did have visual and auditory contact. Birds had ad libitum access to food (Mazuri Small Bird Maintenance Diet; Mazuri, St. Louis, MO, USA), water with vitamins supplemented on alternating days (Monday, Wednesday, Friday; Prime Vitamin Supplement; Hagen, Inc.), a cup containing grit, and a cuttlebone. Additional nutritional supplements included three to five sunflower seeds daily, one superworm (*Zophobas morio*) three times a week, and a mixture of hard-boiled eggs and greens (spinach or parsley) twice a week. The colony rooms were maintained at approximately 20 °C and on a light:dark cycle that followed the natural light cycle for Edmonton, Alberta, Canada.

One bird had previous experience with one operant experiment involving *chick-a-dee* calls but showed no difference in responding in comparison to the naïve birds. The remaining 15 birds had no previous experimental experience with black-capped chickadee-produced *fee-bee* songs or any experimental paradigm.

### Recordings of acoustic stimuli

The following acoustic stimuli were used in our previous published operant study which indicated that male and female chickadees can identify individual females via their song^36^. Stimuli included the songs of six female black-capped chickadees. All females were captured in Edmonton (North Saskatchewan River Valley, 53.53°N, 113.53°W; Mill Creek Ravine, 53.52°N, 113.47°W), Alberta, Canada in January 2010, 2011, 2012, and 2014, and all females were at least one year of age at capture, verified by examining outer tail rectrices^38^. Four females were recorded in Spring 2012 and two females were recorded in Fall 2014. Each recording session lasted approximately 1 h and all recordings took place after colony lights turned on at 08:00, specifically at 8:15. All females were recorded in silence, individually, within their respective colony room cages. Colony room cages were placed in sound-attenuating chambers for recording (1.7 m × 0.84 m × 0.58 m; Industrial Acoustics Company, Bronx, NY). An AKG C 1000S (AKG Acoustics, Vienna, Austria) microphone (positioned 0.1 m above and slightly behind the cage) was connected to a Marantz PMD670 (Marantz America, Mahwah, NJ) digital recorder (16 bit, 44,100 Hz sampling rate) and was used for all recordings. Audio recordings were analyzed and cut into individual files (songs) using SIGNAL 5.03.11 software (Engineering Design, Berkley, CA, USA).

### Acoustic stimuli

For the current study, a total of 150 vocalizations were used as stimuli, these vocalizations were comprised of 25 *fee-bee* songs produced by each of six recorded female chickadees. We ensured that all 150 were of high quality, meaning no audible interference, and all stimuli were bandpass filtered (lower bandpass 500 Hz, upper bandpass 14,000 Hz) using GoldWave version 6.31 (GoldWave, Inc., St. John’s, NL, Canada) in order to reduce any background noise outside of the song stimuli spectrum. For each song stimulus, 5 ms of silence was added to the leading and trailing portion of the vocalization and each stimulus was tapered to remove transients, in addition amplitude was equalized peak to peak using SIGNAL 5.03.11 software. When triggered, stimuli were presented at approximately 75 dB peak SPL as measured by a calibrated Brüel & Kjær Type 2239 (Brüel & Kjær Sound & Vibration Measurement A/S, Nærum, Denmark) sound pressure meter (A-weighting, slow response), a level that corresponds with the natural chickadee vocalizations amplitudes^39–41^. All dB measurements were made at the level of the request perch where birds trigger stimuli and where birds are required to remain for the length of the stimuli and all dB measurements refer to SPL.

### Noise stimuli

**Figure 1:**
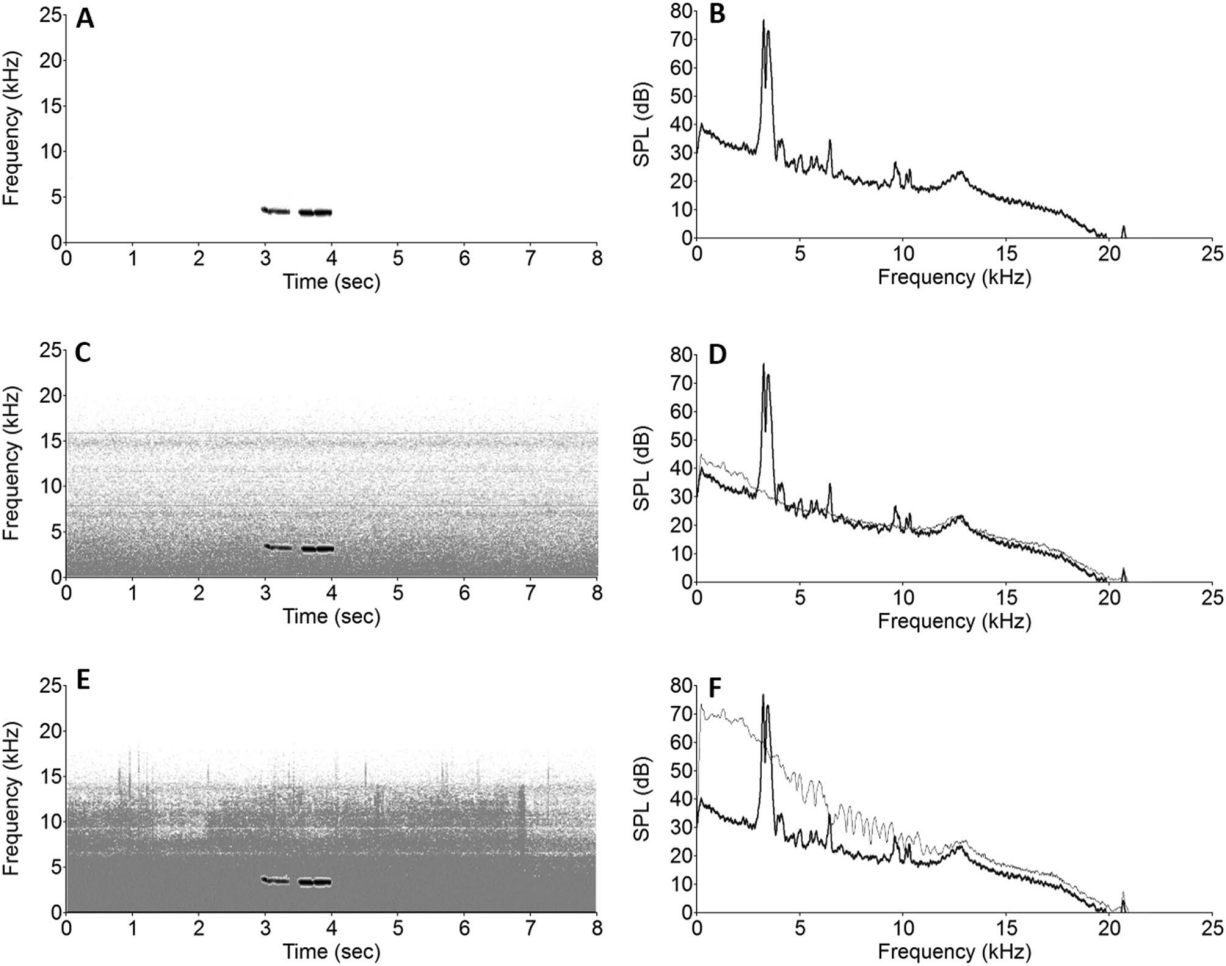
(**A**) Spectrogram of a female *fee-bee* song in silence. (**B**) Power spectrum of female *fee-bee* song in silence . (**C**) Spectrogram of female *fee-bee* song in low noise. (**D**) Power spectrum of female *fee-bee* song (black) in low noise (grey). (**E**) Spectrogram of female *fee-bee* song in high noise. (F) Power spectrum of female *fee-bee* song (black) in high noise (grey).

Anthropogenic noise stimuli were originally created and used by Potvin and MacDougall-Shackleton^42^ and by Potvin, Curcio, Swaddle, and MacDougall-Shackleton^43^. The stimuli were recorded from an urban area in Melbourne, Victoria, Australia and other anthropogenic noise stimuli of various trains, cars, motorcycles, and lawnmowers downloaded from Soundbible.com were used. Within Victoria^44^ and Alberta^45,46^, urban traffic noise averages 60–80 dB SPL. The files used varied in length, with those recorded in Melbourne all being 10 min in length and those downloaded from Soundbible.com varying between 1–10 minutes^42,43^. In total 10 tracks were used with 30 total minutes of noise stimuli. Three anthropogenic noise conditions were used in the study, including Silence (no noise), Low noise (anthropogenic noise stimuli played at ~ 40 dB peak SPL), and High noise (anthropogenic noise stimuli played at ~ 75 dB peak SPL) replicating the variation of traffic noise experienced in urban areas^42,43^. For the Low and High noise conditions the 10 tracks repeated on a randomized loop during data collection (natural light of light/dark cycle) with, thus noise exemplars overlapped songs by chance, to further emulate urban areas. Noise stimuli had natural variations and modulations in frequency and amplitude over the course of the sound files. All dB measurements for noise stimuli included in this study refer to SPL. See Fig. 1 for female song and traffic noise stimuli spectrograms and power spectra.

### Apparatus

**Figure 2:**
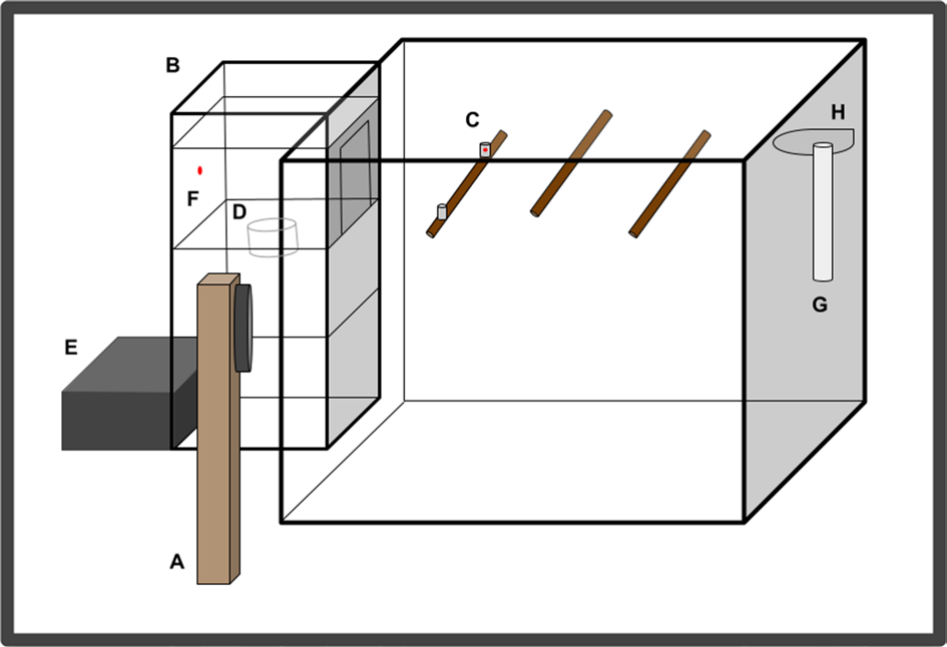
Illustration of the operant conditioning chamber, including: (**A**) speaker, (**B**) automated feeder, (**C**) request perch fitted with infrared photo-beam assembly, (**D**) feeder cup, (**E**) electrical inputs, (**F**) red LED, (**G**) water bottle, (**H**) and cuttlebone. Also shown is the feeder opening, and additional perches. To simplify, the sketch the front and floor of the chamber, and the enclosure's acoustic lining are not included.

For the duration of the experiment, birds were housed individually in modified colony room cages (30 × 40 × 40 cm; described above) which were placed inside a ventilated, sound-attenuating operant chamber. See Fig. 2 for illustration of operant conditioning chamber. All chambers were lit with a full spectrum LED bulb (3 W, 250 lm E26, Not-Dim, 5000 K; Lohas LED, Chicago, IL, USA), and maintained the natural light:dark cycle for Edmonton, Alberta. Each cage within each operant chamber contained two perches and an additional perch fitted with an infrared sensor (i.e., the request perch). See Fig. 2C. Each cage also contained a water bottle, grit cup, and cuttlebone See Fig. 2G-2H. Birds had ad libitum access to water (with vitamins supplemented on alternating days; Monday, Wednesday, Friday), grit, and cuttlebone and were provided two superworms daily (a morning and afternoon worm). An opening (11 × 16 cm) located on the left side of the cage allowed the birds to access a motorized feeder, with a red LED light, and equipped with an infrared sensor^47^. See Fig. 2B,D–F. The purpose of the sensor was so that food was only available as a reward for correct responses to auditory stimuli during the operant discrimination task. We should note that performance of the discrimination task is required for access to food and thus maintains motivation. For operation and data collection, a personal computer connected to a single-board computer^48^ scheduled trials and recorded responses to stimuli. Stimuli were played from a personal computer hard drive through a Cambridge Integrated Amplifier (model A300 or Azur 640A; Cambridge Audio, London, England). Data is downloaded once a day in order to reduce stress on subjects as all equipment must be tested following download, requiring contact with subjects. Stimuli played in the chamber through a Fostex full-range speaker (model FE108 Σ or FE108E Σ; Fostex Corp., Japan; frequency response range 80–18,000 Hz) located beside the feeder. See Sturdy and Weisman^49^ for a detailed description of the apparatus. See Fig. 2 for an illustration of the operant conditioning chamber set-up.

### Procedure

#### Operant conditioning

Our current operant conditioning go/no-go set-up is used to understand how birds perceive auditory stimuli. By training the birds to respond to particular stimuli and withhold responding to other stimuli we can compare responses to both types of stimuli. The go/no-go paradigm requires the birds to learn which stimuli require correct responses (go), providing reinforcement (food), and which stimuli require birds to withhold responding (no-go), resulting in the avoidance of punishment (lights out).

The current study follows nine stages, after learning to use the operant conditioning set-up, birds then go through Non-differential training (stage 1) where they will be exposed to all stimuli that will be used in the experiment and to ensure that the birds respond to the stimuli equivalently. Then birds complete Discrimination training (stage 2) where birds on two categories of sounds. One category is rewarded, the other category is punished. Then the Discrimination-85 (stage 3) phase prepares birds for future trials where there is no reward nor punishment. After this point, birds will follow three series (Silence; Low; High) of Discrimination-85 with noise (stage 4, 6, 8) and a corresponding Probe with noise (stage 5, 7, 9), meaning that that each subject will repeated the two discrimination tasks three times with different noise conditions, with the order of noise conditions randomized among individuals. The detailed procedures for each stage are described in the following.

#### Non-differential training

The purpose of Non-differential training is to engender a high level of responding on all trials, across all stimuli. Once a bird learned to use the request perch fitted with a sensor as well as learned to use the feeder to obtain food then Pretraining began. During Pretraining, birds were trained to respond to a 1 s tone (1,000 Hz) in order to receive access to food. Pretraining occurred over an approximately 15-day period in order to allow acclimatization to the chamber, feeder, and speaker. Following Pretraining was Non-differential training. During Non-differential training, birds received food for responding to all *fee-bee* song stimuli. All trials began when a bird landed on the request perch and remained on the perch for between 900–1100 ms, at which point a randomly-selected song stimulus played. Songs were presented in random order from trial to trial until all 150 stimuli had been triggered and played without replacement; once all 150 stimuli were played, a new random sequence initiated. In the event that the bird left the request perch during a stimulus presentation, the trial was deemed interrupted, and resulted in a 30 s lights out of the operant chamber. If the bird entered the feeder within 1 s after the stimulus (any stimulus) was played, it was given 1 s access to food, followed by a 30 s intertrial interval. If a bird remained on the request perch during the stimulus presentation and the 1 s following the completion of the stimulus, then the bird received a 60 s intertrial interval with the lights on. Birds continued on Non-differential training until they completed six 450-trial blocks at ≥ 60% responding on average to all stimuli, at least four 450-trial blocks at ≤ 3% difference in responding to future rewarded versus future unrewarded Discrimination stimuli, at least four 450-trial blocks at ≤ 3% difference in responding to future rewarded versus unrewarded Discrimination stimuli. Then following a day of free feed (during which birds had ad libitum access to a food cup) birds completed a second round of Non-differential training in which they completed at least one 450-trial block that met each of the above requirements. A 450-trial block consisted of the bird experiencing each of the 150 stimuli three times. For the current study the average time to complete Non-differential training ranged from 10 to 41 days (*M* = 21.43, *SD* = 9).

#### Discrimination training

Discrimination training procedures included only 114 out of the 150 training stimuli that were previously presented in non-differential training, and responses to these stimuli were now differentially reinforced. Specifically, correct responses to half of the stimuli (“rewarded stimuli”, S+) were positively reinforced with 1 s access to food, and incorrect responses to the other half (“unrewarded stimuli”, S−) were instead punished with a 30-s intertrial interval of lights off within the operant chamber. In regard to criterion, Discrimination training continued until a bird completed six 342-trial blocks with a discrimination ratio between their respective S+ and S− of greater than 0.80 with the last two blocks being consecutive. For discrimination ratio calculations see Response Measures below.

The current subjects were randomly assigned to either a True category discrimination group (*n* = 10) or Pseudo category discrimination group (*n* = 6). Furthermore, chickadees in the True category discrimination group were divided into two subgroups: (a) True 1 (*n* = 5; three females and two males) discriminated between 57 rewarded *fee-bee* songs produced by three individual female chickadees (S+) and 57 unrewarded *fee-bee* songs produced by another three individual female chickadees (S−); and (b) True 2 (*n* = 5; two females and three males) discriminated between the same songs with opposite rewards, properly, the 57 rewarded (S+) *fee-bee* songs were the S− from True 1 and the 57 unrewarded (S−) *fee-bee* songs were the S+ from True 1. For birds in the True category discrimination the average number of blocks completed per day for Discrimination training ranged from 2.4–4.4 blocks (3.3 ± 0.7 blocks).

In similitude, the Pseudo category discrimination group was divided into two subgroups: (a) Pseudo 1 (*n* = 3; two females and one male) discriminated between 57 randomly-selected rewarded (S+) *fee-bee* songs and 57 randomly-selected unrewarded (S−) *fee-bee* songs; and (b) the second subgroup Pseudo 2 (*n* = 3; one female and two males) discriminated between the same songs with opposite rewards, meaning, the 57 rewarded (S+) fee-bee songs were the S− from Pseudo 1 and the 57 unrewarded (S−) fee-bee songs were the S+ from Pseudo 1 (S+) *fee-bee* songs and 57 randomly-selected unrewarded (S−) *fee-bee* songs. To explicate, the purpose of the two Pseudo groups was to include a control in which subjects are required to memorize each vocalization independent of the producer rather than be trained to categorize songs according to individual chickadees as the True groups have been. All birds remained in their respective groups (True 1 and 2; Pseudo 1 and 2) for the duration of the study. For birds in the Pseudo category discrimination the average number of blocks completed per day for Discrimination training ranged from 3.3–6.1 blocks (4.34 ± 1.2 blocks).

#### Discrimination-85 phase

Discrimination-85 was identical to the above Discrimination training except that rewarded songs were reinforced with a reduced probability, P = 0.85. Therefore, for 15% of trials when a rewarded stimulus was played and a bird correctly responded, no access to food was triggered. Instead, a 30 s lights on intertrial interval occurred. The change in reinforcement occurs in order to prepare birds for Probe trials in which novel song stimuli were neither rewarded with access to food nor unrewarded with a lights out, instead nothing occurs. Discrimination-85 continued until birds completed two consecutive 342-trial blocks with a discrimination ratio of at least 0.80.

#### Discrimination-85 phase with noise

All subjects followed three series (Silence; Low; High) of Discrimination-85 with noise and a corresponding Probe with noise and the order of noise stimuli was randomly-selected for each bird. Discrimination-85 with noise was identical to the Discrimination-85 phase except one of the three noise stimuli conditions (Silence; Low noise, 40 dB SPL; High noise, 75 dB SPL) was played over the song stimuli. The noise stimuli condition was randomly-selected for each bird. Each bird went through three series of Discrimination-85 with noise (Silence; Low; High) until reaching criteria: two consecutive 342-trial blocks with a discrimination ratio of at least 0.80. Here, we were interested in how the addition of noise would impact discrimination between rewarded and unrewarded female song stimuli.

### Probe phase with noise

Following each Discrimination-85 phase with noise was a corresponding Probe phase with noise. During Probe the reinforcement contingencies from Discrimination-85 were maintained. In addition to the 114 stimuli from Discrimination training, this stage included 12 novel *fee-bee* songs (i.e., Probe stimuli), two from each of the six individual females. For True groups, six of these novel songs were categorized as P + and the other six as P-, based on whether they were produced by the same birds as the S+ or the S− training stimuli. For Pseudo groups, the novel songs were not assigned to categories. For both groups, the 12 novel stimuli were neither rewarded (no food access) nor unrewarded (no lights out). The birds completed six 126-trial blocks in which the 114 familiar discrimination stimuli repeated once per block and the 12 probe sequences played once per block. In addition, one of the three noise stimuli conditions (Silence; Low noise, 40 dB SPL; High noise, 75 dB SPL) was played over the song stimuli, and each bird went through three series of Probe with noise (Silence; Low; High) which corresponded with the birds previous Discrimination-85 phase with noise condition. Thus, all birds completed all three Discrimination-85 phases with noise conditions followed by the corresponding Probe with noise conditions, and the order of noise stimuli condition was randomly-selected for each bird. In Probe phases we are interested if subjects can categorize novel stimuli to previously rewarded or unrewarded female birds.

#### Response measures

For each 342-block trial during training (Discrimination-85 with noise; Probe with noise), proportion response was calculated (R + /(N-I)): R + represents the number of trials in which the bird went to the feeder, N represents the total number of trials, and I represents the number of interrupted trials in which the bird left the perch before the entire stimulus played. For Discrimination training and the Discrimination-85 phase, a discrimination ratio was calculated by dividing the mean proportion response to all S+ stimuli by the mean proportion response to S+ stimuli plus the mean proportion response to S− stimuli. A discrimination ratio = 0.50 specifies equal response to rewarded (S+) and unrewarded (S−) stimuli, a discrimination ratio = 1.00 specifies a perfect discrimination between S+ and S− stimuli. We also collected data regarding the number of blocks and days per stage (Discrimination training; Discrimination-85 training with noise) in order to examine the latency of discrimination learning.

### Statistical analyses

All statistical analyses were conducted using SPSS (Version 20, Chicago, SPSS Inc.). In order to compare the number of trials needed to reach criterion and the discrimination ratios between True and Pseudo groups for Discrimination Training we conducted an analysis of variance (ANOVA). For Discrimination-85 with noise (Silence, Low noise, High noise), an ANOVA was conducted to compare the number of trials needed to reach criterion and the discrimination ratios between True and Pseudo groups. We also conducted *post-hoc* tests in order to reveal any sex differences between groups.

And for Discrimination-85 with noise and Probes with noise repeated measures ANOVA was conducted to compare proportion response to training stimuli and probe stimuli between True groups and Pseudo groups. Lastly, we conducted *post-hoc* tests in order to reveal any differences in the number of trials to reach criterion during Discrimination training and to Discrimination-85 with noise.

### Ethics declaration

Throughout the experiment, birds remained in the testing apparatus to minimize the transport and handling of each bird. One male and two female subjects died from natural causes during operant training. Following the experiment, healthy birds were returned to the colony room for use in future experiments.

All procedures were conducted in accordance with the Canadian Council on Animal Care (CCAC) Guidelines and Policies with approval from the Animal Care and Use Committee for Biosciences for the University of Alberta (AUP 1937), which is consistent with the Animal Care Committee Guidelines for the Use of Animals in Research. Birds were captured and research was conducted under an Environment Canada Canadian Wildlife Service Scientific permit (#13-AB-SC004), Alberta Fish and Wildlife Capture and Research permits (#56,066 and #56,065), and the City of Edmonton Parks permit. All methods are reported in accordance with ARRIVE guidelines.

## Results

### The differences of discrimination ratios between True and Pseudo groups during Discrimination Training

**Figure 3:**
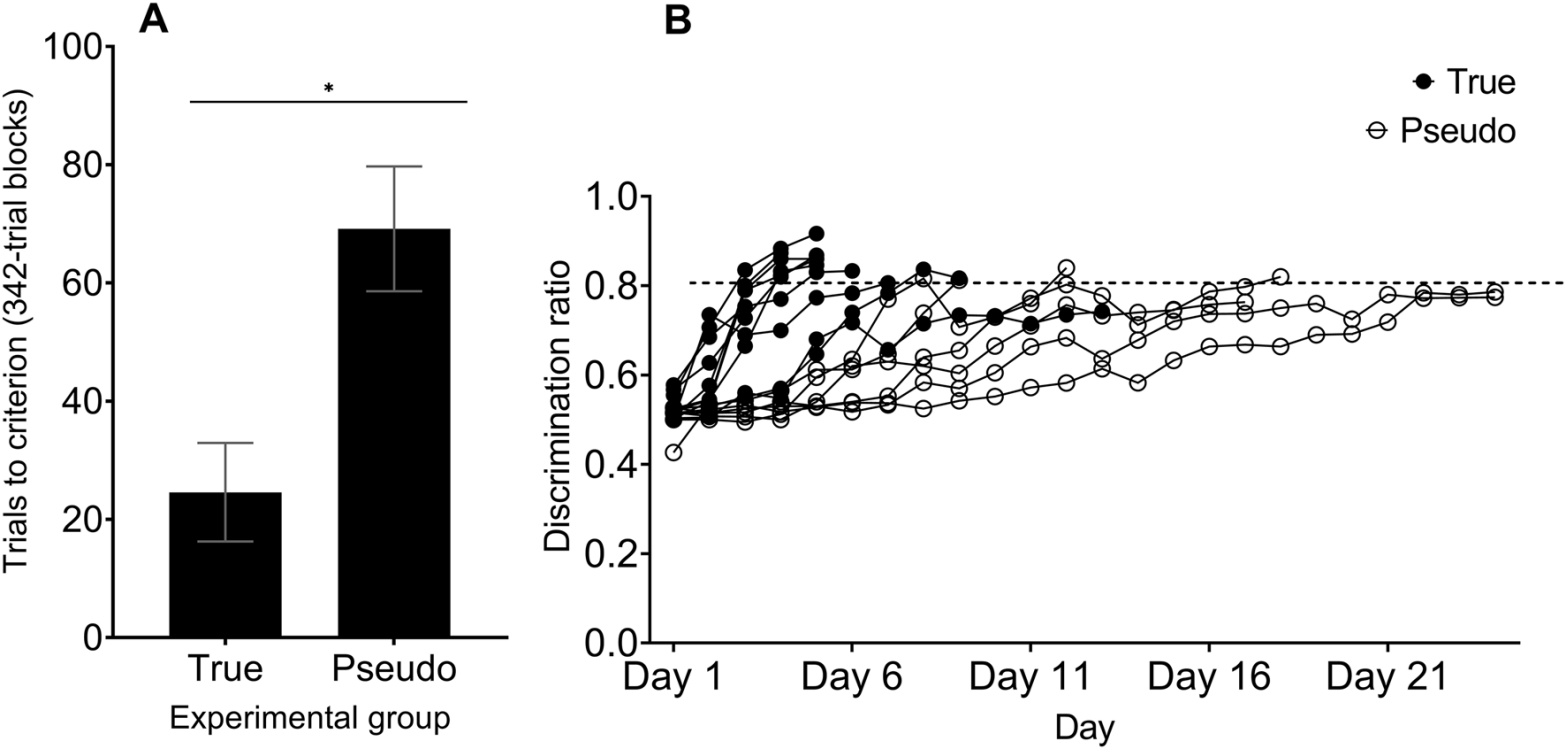
(**A**) Trials to criterion by True groups and Pseudo groups in Discrimination training. The following difference was significant (indicated by asterisk): True groups vs. Pseudo groups in Discrimination training (ANOVA, F_1,12_ = 11.801, *p* = 0.005). Error bars represent standard error. (**B**) Average discrimination ratio for all True (*n* = 10) and Pseudo (*n* = 6) birds by number of days during Discrimination training. Birds completed Discrimination training via six 342-trial blocks with a discrimination ratio greater than 0.80 (dashed line) with the last two blocks being consecutive.

Results showed that for Discrimination training, True group birds reached criterion (i.e., learned to discriminate in fewer sessions) significantly faster (22.985 ± 8.342 blocks; 69.167 ± 10.552 blocks) than did Pseudo group birds based on discrimination ratios (*F*_1,12_ = 11.801, *p* = 0.005, η_p_^2^ = 0.496). See Fig. 3A for trials to criterion. On average, the number of days to reach criterion for True birds ranged from 4–24 days (7.5 ± 5.9 days) and on average Pseudo birds reached criterion in 9–24 days (18.4 ± 6 days). See Fig. 3B for discrimination ratios by day. There was no significant difference in sessions to criterion between the two sex (*F*_1,8_ = 0.294, *p* = 0.598, η_p_^2^ = 0.024). Further Tukey’s post hoc analysis showed no significant difference between True groups 1 and 2 (*p* = 0.384), and no significant difference between Pseudo 1 and 2 (*p* = 0.125).

### The differences of discrimination ratios between True and Pseudo groups during Discrimination-85 with noise

**Figure 4:**
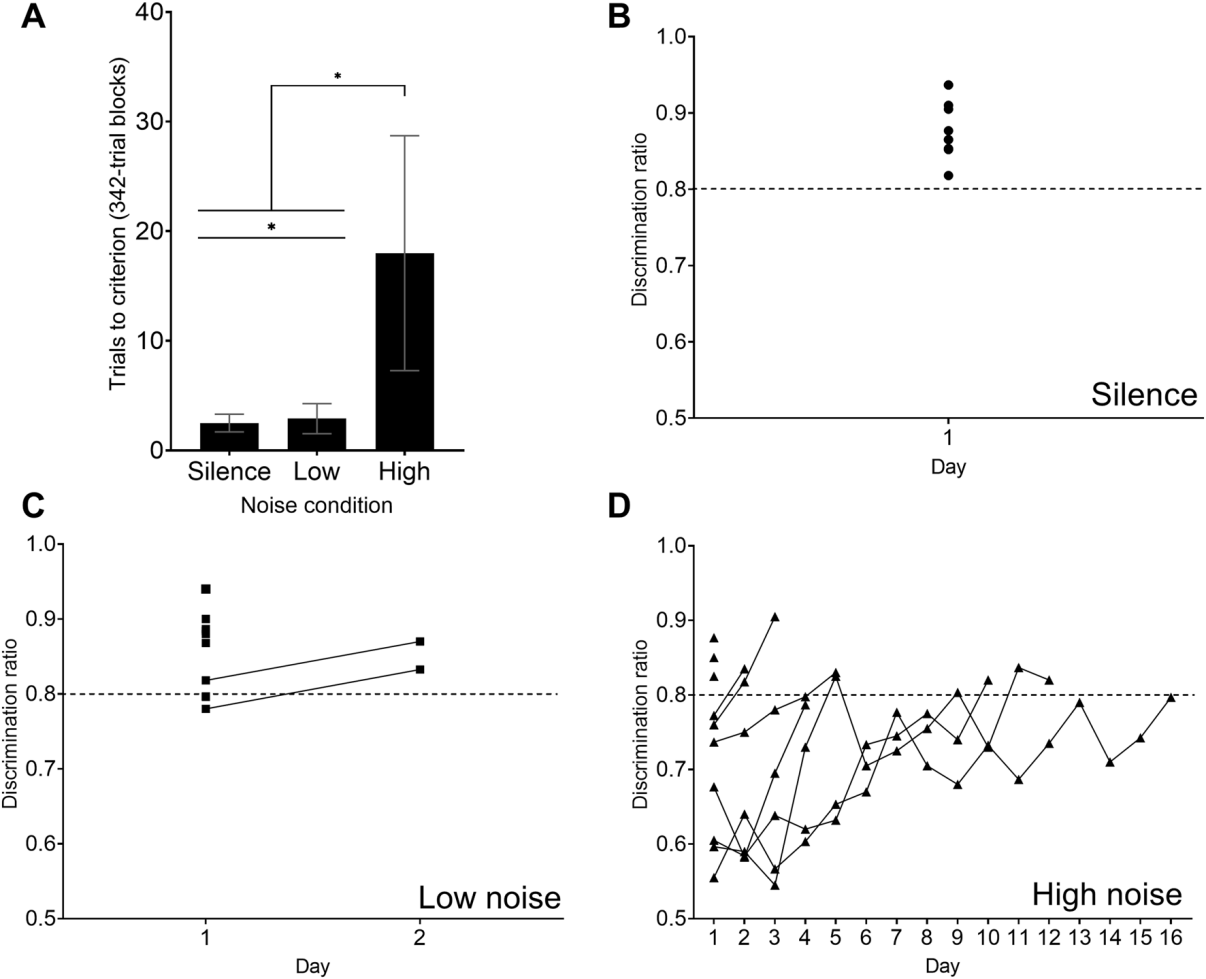
(**A**) Trials to criterion for Discrimination-85 with noise. The following differences were significant (indicated by asterisk): High noise condition vs. Silence (*p* < 0.001), High noise condition vs. Low noise condition (*p* < 0.001), and Low noise vs. Silence condition (*p* = 0.002). Error bars represent standard error. (**B**) Average discrimination ratio for all True (*n* = 10) birds by number of days during Discrimination-85 with silence. Birds completed Discrimination-85 with noise via two consecutive 342-trial blocks with a discrimination ratio of at least 0.80 (dashed line). (**C**) Average discrimination ratio for all True (*n* = 10) birds by number of days during Discrimination-85 with low noise. (**D**) Average discrimination ratio for all True (*n* = 10) birds by number of days during Discrimination-85 with high noise.

There was a significant difference between the three noise conditions (Silence, 2.437 ± 0.388 blocks; Low, 9.708 ± 1.265 blocks; High, 43.896 ± 5.031 blocks) of Discriminatio-85 training based on discrimination ratios, (*F*_2,16_ = 50.706, *p* < 0.001, η_p_^2^ = 0.864). Specifically, there were significant differences in the rate of acquisition for the High noise condition compared to the Silence (*p* < 0.001) and Low noise conditions (*p* < 0.001), as well as between the Low noise and Silence conditions (*p* = 0.002). See Fig. 4A for trials to criterion.

Results for Discrimination-85 with Silence showed no significant difference in trials to criterion between True and Pseudo group birds based on discrimination ratios (*F*_1,12_ = 0.450, *p* = 0.835, η_p_^2^ = 0.004) as well as no differences by sex (*F*_1,12_ = 0.725, *p* = 0.411, η_p_^2^ = 0.060). A follow-up Tukey’s post hoc analysis also showed no significant difference between True groups 1 and 2 (*p* = 1.000), and no significant difference between Pseudo 1 and Pseudo 2 (*p* = 0.504). In terms of days to reach criterion for True birds, Discrimination-85 with Silence was completed in one day (1.0 ± 0.0 days). See Fig. 4B for trials to criterion by day. For Pseudo birds, Discrimination-85 with Silence was completed in 1–3 days (1.8 ± 1.1 days).

For Discrimination-85 with Low noise, results showed that True group birds were able to reach criterion significantly faster than did Pseudo group birds based on discrimination ratios (*F*_1,12_ = 15.501, *p* = 0.002, η_p_^2^ = 0.564), meaning True birds learned to discriminate more quickly than Pseudo birds in the presence of Low noise. In addition, there was no significant difference in trials to criterion by sex (*F*_1,12_ = 0.656, *p* = 0.222, η_p_^2^ = 0.121). And Tukey’s post hoc analysis also showed no significant difference between True groups 1 and 2 (*p* = 0.220), and no significant difference between Pseudo 1 and 2 (*p* = 0.368). For True birds, Discrimination-85 with Low noise was completed in 1–2 days (1.2 ± 0.4 days). See Fig. 4C for trials to criterion by day. For Pseudo birds, Disicrimation-85 with Low noise was completed in 1–9 days (4.8 ± 4.0 days).

Lastly, results for Discrimination-85 with High noise, revealed that True group birds reached criterion significantly faster than did Pseudo group birds based on discrimination ratios (*F*_1,12_ = 10.000 *p* = 0.008, η_p_^2^ = 0.455), again meaning that True birds learned to discriminate between individuals faster than Pseudo birds when in the presence of High noise. There was a significant difference in trials to criterion by sex (*F*_1,12_ = 9.173, *p* = 0.010, η_p_^2^ = 0.433). Tukey’s post hoc analysis showed no significant difference between True groups 1 and 2 (*p* = 0.326), but a significant difference between Pseudo 1 and 2 (*p* = 0.008). The Tukey’s post hoc analysis also showed no significant difference by sex for True groups 1 and 2 (*p* = 0.840), but a significant difference between Pseudo 1 and Pseudo 2 (*p* = 0.001) with females learning the discrimination faster than males. For True birds, Discrimination-85 with High noise was completed in 1–16 days (5.4 ± 5.4 days). See Fig. 4D for trials to criterion by day. For Pseudo birds, Disicrimation-85 with High noise was completed in 2–39 days (19.2 ± 14.8 days).

### The differences of proportion response in True and Pseudo groups during Discrimination-85 with noise

**Figure 5:**
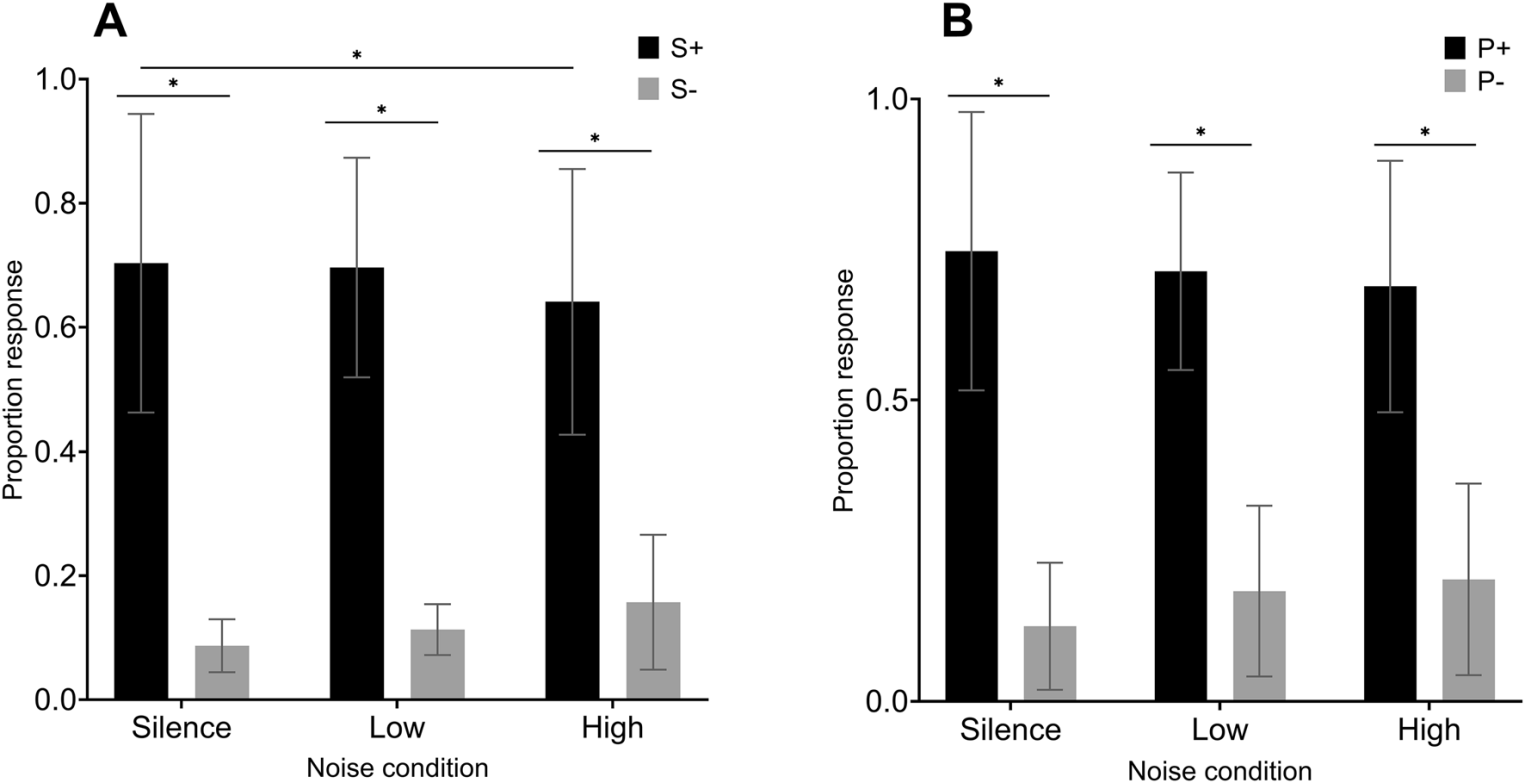
(**A**) Proportion response by True groups in Discrimination-85 with noise. S+ representing responses to rewarded stimuli and S− representing responses to unrewarded stimuli. The following differences were significant (indicated by asterisk): Silence (S+ vs. S−; p < 0.001, Low noise (S+ vs. S−; p < 0.001), High noise (S+ vs. S−; p < 0.001), and Silence vs. High (S+ vs. S+); p 0.017). Error bars represent standard error. (**B**) Proportion response by True groups in Probe trials with noise. P + and P- were based on whether stimuli was produced by the same birds as the S+ rewarded or the S− unrewarded stimuli. The following differences were significant (indicated by asterisk): Silence (S+ vs. S−; p < 0.001, Low noise (S+ vs. S−; p < 0.001), and High noise (S+ vs. S−; p < 0.001). Error bars represent standard error.

In Discrimination-85 with noise, proportion response in True groups differed across the six stimulus types: rewarded stimuli during Silence, unrewarded stimuli during Silence rewarded stimuli during Low noise, unrewarded stimuli during Low noise, rewarded stimuli during High noise, and unrewarded stimuli during High noise (F_1,8_ = 92.498, *p* < 0.001, η_p_^2^ = 0.920). See Fig. 5A for proportion response. Tukey’s post-hoc analysis revealed a significant difference in proportion response between rewarded (S+) and unrewarded (S−) stimuli during Silence, Low, and High noise (*p*s < 0.001). We also found no difference in proportion response between rewarded stimuli (S+) during Silence vs. Low noise (*p* = 0.780), or Low vs. High noise (*p* = 0.164) but there was a significant difference between rewarded stimuli (S+) during Silence vs. High noise, based on proportion response (*p* = 0.017). Lastly, there were no significant differences in proportion response between unrewarded stimuli (S−) during any noise condition (*p*s > 0.060).

In Discrimintion-85 training with noise, proportion response in Pseudo groups also differed across the six stimulus types: rewarded stimuli during Silence, unrewarded stimuli during Silence rewarded stimuli during Low noise, unrewarded stimuli during Low noise, rewarded stimuli during High noise, and unrewarded stimuli during High noise (F_1,4_ = 30.904, *p* = 0.005, η_p_^2^ = 0.885). For Pseudo birds, Tukey’s post-hoc analyses revealed a significant difference in proportion response between rewarded (S+) and unrewarded (S−) stimuli during Silence (*p* = 0.022), no difference between rewarded (S+) and unrewarded (S−) stimuli during Low noise (*p* = 0.091), but a significant difference between rewarded (S+) and unrewarded (S−) stimuli during High noise (*p* = 0.016). Analyses also revealed no difference in proportion response between rewarded stimuli (S+) during Silence and High noise (*p* = 0.157), and Low and High noise (*p* = 0.609) but there was a significant difference between rewarded stimuli (S+) during Silence and Low noise, based on proportion response (*p* = 0.036). Lastly, there was no significant difference in proportion response between unrewarded stimuli (S−) during any noise condition (*p*s > 0.182).

### The differences of proportion response in True and Pseudo groups during Probe with noise

In Probe with noise, proportion response in True groups differed significantly across the six stimulus types: rewarded stimuli during Silence, unrewarded stimuli during Silence rewarded stimuli during Low noise, unrewarded stimuli during Low noise, rewarded stimuli during High noise, and unrewarded stimuli during High noise (F_1,8_ = 94.601, *p* < 0.001, η_p_^2^ = 0.922). See Fig. 5B for proportion response. Tukey’s post-hoc analysis showed a significant difference in proportion response between rewarded (S+) and unrewarded (S−) stimuli during Silence, Low, and High noise (*p*s < 0.001). We also found no difference in proportion response to rewarded stimuli (S+) between any noise condition (*p*s > 0.337), or any difference in proportion response to unrewarded stimuli (S−) between any noise condition (*p*s > 0.211).

## Discussion

Based on discrimination ratios, our predictions were in line with our results which suggest that as noise level increased, learning performance between individual females via their song decreased. Low noise and high noise were detrimental to learning the discrimination (i.e., impaired discrimination performance), with high noise impairing discrimination more than low noise. However, only in Silence did True group birds learn the discrimination significantly faster than Pseudo groups birds, suggesting True category discriminations were easier to learn versus memorizing randomly-selected rewarded songs. High or low noise learning was disrupted in both True and Pseudo groups. Thus, even a low-level noise of 40 dB SPL impacted the bird’s ability to discriminate between individuals. We also saw no differences by sex, suggesting that both sexes are being impacted by noise and possibility that both sexes perceive female *fee-bee* song in a similar way.

At the Discrimination-85 with noise training stage, birds had already learned the discrimination and responded differentially to the six stimuli types: (1) rewarded stimuli during Silence, (2) unrewarded stimuli during Silence, (3) rewarded stimuli during Low noise, (4) unrewarded stimuli during Low noise, (5) rewarded stimuli during High noise, and (6) unrewarded stimuli during High noise. Proportion response data for Discrimination-85 with noise shows that both True and Pseudo group birds responded to rewarded and unrewarded *fee-bee* song stimuli consistently across noise types but differed in their responding by noise type. Meaning that for Silence, Low, and High noise, birds responded significantly more to rewarded compared to unrewarded, thus learning their discrimination which is also demonstrated by trials to criterion for all noise conditions. However, when looking at proportion response to only rewarded stimuli across conditions for True groups, High noise had significantly less response compared to the Silence group, further indicating that noise was detrimental to discriminating between individual female songs. In addition, responding to unrewarded stimuli across noise conditions increased as noise increased, albeit not significantly. Lastly, Discrimination-85 with noise and Probe with noise percent response data surprisingly suggests that True birds did learn to generalize responding in all noise conditions, demonstrating that birds did transfer their learning of specific female individual song to novel song stimuli. And birds in Silence, Low, or High noise conditions did not differ in responding across reward stimuli or across unrewarded stimuli, indicating that responding was maintained across noise conditions.

The songs used in the current study were produced and recorded in the relative silence of a sound attenuating chamber in a laboratory. These recorded songs were then presented to the subjects with the addition of anthropogenic noise. Previous research has shown that black-capped chickadees require prior experience with noise to adjust their vocalizations in response to noise^33^. Conceivably, the same is true for accurately perceiving songs in anthropogenic noise and over time, or through multiple sessions over time, birds would improve their discrimination between individuals. In addition, different results may have been expected or observed if songs recorded in anthropogenic noise were used. Songs that have naturally been shifted in their amplitude or frequency to be heard over noise may no longer show masking effects. Although, a past study on great tits found that masking still impacted vocalizations produced in noise^13^. Tits were recorded in a lab setting with anthropogenic noise present, and recordings showed that the amplitude of calls increased. However, when the modified calls were used as stimuli in a playback field study, traffic noise masked the modified alarm calls.

In terms of noise stimuli, the current study used a combination of recorded anthropogenic noise stimuli as well as other anthropogenic noise stimuli (i.e., trains, cars, motorcycles, lawnmowers). We should note that the random combination of noise stimuli is not identical to what a bird would typically experience in an urban environment. A recent study has found that zebra finches do not increase their song frequencies as adults when exposed to natural anthropogenic noise during the sensorimotor learning period^50^. A similar result has been found for synthetic noise with zebra finches^43^ and great tits^51^, however, another study has suggested that artificial noise mimicking the spectral shape of noise does impact the development of song in white-crowned sparrows (*Zonotrichia leucophrys*)^52^. It is possible that the generalization to individual female *fee-bee* songs that we observed in the current study was due to behavioral plasticity, but we should consider the combination of anthropogenic stimuli (i.e., noise recorded in an urban area; other anthropogenic noise; See Noise Stimuli) used in the current study.

While individual recognition was impaired by noise in the current study, we may be also observing the result of impaired detection^53,54^, suggested by the differences in responding based on noise condition. A past lab operant conditioning go/no-go study with great tits found that signal detection is impacted by anthropogenic noise^52^. The study used multiple independent masking effects (urban noise, woodland noise, dawn chorus) and found that auditory thresholds during noise, both urban and woodland, required louder signals compared to no-noise/no-masking effects. In addition, birds were better able to detect signals with a narrow frequency range vs. a wide frequency range in no-noise, urban, and woodland conditions. Perhaps the birds in the current study were experiencing a similar masking effect and impaired detection/recognition. Another operant conditioning go/no-go task using great tits unexpectedly found low critical masking ratios at high frequencies, suggesting that great tits can perceive high-frequency signals in order to communicate in the presence of white noise^54^, and that the songs in the current study could also be masked. In a similar vein, the reduced learning performance in the presence of noise (See Fig. 4) that we are observing in the current study could be due also to other factors such as distraction (i.e., distracted prey hypothesis; See Introduction)^15^. Overall, these findings highlight the potential impacts of ever-increasing anthropogenic noise on wildlife. In particular, we highlight the impact on perception of auditory signals. Research has demonstrated that noise can mask communication between and within species, yet some species thrive in the city and show phenotypic differences in behavior, physiology, and morphology when compared to their rural conspecifics. Previous studies have shown that phenotypic and environmental variation are correlated^55^ and their relationship is reflected by distinct mechanisms such as vocal plasticity^56^. Studies of nightingales (*Luscinia megarhynchos*) and great tits demonstrate that birds adjust song amplitude^9,13^ in response to background noise. Black-capped chickadees have been found to sing at a higher pitch with increases in anthropogenic noise^22^.

These and other findings suggest that birds are modifying their vocalizations as a result of noise in order to communicate with conspecifics^57,58^. However, how are the receivers, both conspecific and heterospecific, of these modified signals perceiving these modified vocalizations? And how does signal detection impact these perceptions? For future studies, black-capped chickadees serve as an ideal subject given that they are both urban and rural birds, and further exploring the differences between these birds can aid in understanding how environmental pressures and evolutionary responses change vocalizations in production and perception = . For example, what differences do we see in urban vs. rural birds in terms of behavior, quality, and perceptual abilities. Behavioral traits such as aggressiveness and boldness have also been linked to urbanization and gradients of anthropogenic noiseyy^59,60^. A bolder individual may be drawn to urban areas as they are better equipped to survive there. Overall, we find an abundance of research topics related to noise and animal communication left to explore in which chickadees serve as an ideal model to investigate the perception abilities and the effects of urbanization.

## Data Availability

Analyses reported in this article can be reproduced using the data provided by https://osf.io/wa62x/?view_only=4de5e8d354bf4ac59e56ce8231b46752.
